# Impacts of tai chi exercise on functional fitness in community-dwelling older adults with mild degenerative knee osteoarthritis: a randomized controlled clinical trial

**DOI:** 10.1186/s12877-021-02390-9

**Published:** 2021-07-31

**Authors:** Po-Yin Chen, Chen-Yi Song, Hsin-Yen Yen, Pi-Chu Lin, Su-Ru Chen, Liang-Hsuan Lu, Chen-Li Tien, Xin-Miao Wang, Chueh-Ho Lin

**Affiliations:** 1 Department of Physical Therapy and Assistive Technology, National Yang-Ming University, Taipei, Taiwan, Republic of China; 2 Department of Long-Term Care, National Taipei University of Nursing and Health Sciences, Taipei, Taiwan, Republic of China; 3 School of Gerontology Health Management, College of Nursing, Taipei Medical University, Taipei, Taiwan, Republic of China; 4 Master Program in Long-Term Care, College of Nursing, Taipei Medical University, Taipei, Taiwan, Republic of China; 5 Post-Baccalaureate Program in Nursing and School of Nursing, College of Nursing, Taipei Medical University, Taipei, Taiwan, Republic of China; 6 Faculty of Humanities, Zhejiang Dong Fang Polytechnic Collage, Wenzhou, China; 7 Master Program in Long-Term Care, College of Nursing, Taipei Medical University, 250 Wu-Xing Street, 11031 Taipei, Taiwan, Republic of China; 8 Center for Nursing and Healthcare Research in Clinical Practice Application, Wan Fang Hospital, Taipei Medical University, 250 Wu-Xing Street, 11031 Taipei, Taiwan, Republic of China

**Keywords:** Older adults, Tai chi exercise, Dunctional fitness, Randomized controlled clinical trial

## Abstract

**Background:**

Degenerative osteoarthritis (OA) often leads to pain and stiffness of the affected joints, which may affect the physical performance and decrease the quality of life of people with degenerative knee OA. Compared to traditional exercise, tai chi is a safe exercise with slow movements which can facilitate physical functioning and psychological well being, and might be suitable for improving the physical activities of older adults with knee OA. Therefore, this study investigated the impacts of tai chi exercise on the functional fitness of community-dwelling older adults with degenerative knee OA.

**Methods:**

Sixty-eight community-dwelling older adults with knee OA were recruited from the local community to participate in this randomized controlled clinical trial. All subjects were randomly assigned to either an TCE group that practiced tai chi exercise (TCE) (*n* = 36) or a control group (CON) (*n* = 32) that received regular health education programs twice per week for 12 weeks. Outcome measurements were determined using functional fitness tests before and after the intervention, including a 30-s chair stand (number of repeats), 30-s arm-curl (number of repeats), 2-min step (number of steps), chair sit-and-reach (reaching distance, cm), back-scratch flexibility (distance between hands, cm), single-leg stand (time, s), functional reach (reaching distance, cm), 8-foot up-and-go (time, s), and 10-m walk tests (time, s). Pre-post comparisons of functional fitness were analyzed using the ANCOVA test with SPSS software version 18.0.

**Results:**

Results revealed that participants’ functional fitness in the TCE group had significantly higher adjusted mean post-tests scores than that in the CON group after the intervention, including the 8-foot up-and-go (s) (mean difference [MD]=-2.92 [-3.93, -1.91], *p* = 2.39*10^− 7^), 30-s arm curl (MD = 4.75 (2.76, 6.73), *p* = 1.11*10^− 5^), 2-min step (MD = 36.94 [23.53, 50.36], *p* = 7.08*10^− 7^), 30-s chair stand (MD = 4.66 [2.97, 6.36], *p* = 6.96*10^− 7^), functional-reach (MD = 5.86 [3.52, 8.20], *p* = 4.72*10^− 6^), single-leg stand with eyes closed (MD = 3.44 [1.92, 4.97], *p* = 2.74*10^− 5^), chair sit-and-reach (MD = 3.93 [1.72, 6.15], *p* = 0.001), and single-leg stand with eyes opened (MD = 17.07 [6.29, 27.85], *p* = 0.002), with large effect sizes (η²=0.14 ~ 0.34).

**Conclusions:**

Community-dwelling older adults with knee OA in the TCE group had better functional fitness performances after the 12-week tai chi intervention than those receiving only health education.

## Background

Degenerative osteoarthritis (OA) is progressive wear and tear of joint cartilage that increases in prevalence with age. It is estimated that 10 %~20 % of people aged over 60 years suffer from OA [1]. Among all affected joints, knee OA accounts for more than 83 % of the total disease burden [2]. Knee OA is characterized by joint pain and stiffness and causes physical disability, affects the quality of life (QOL), and decreases one’s working ability [3]. The annual expense of treating OA was estimated to be US$15 billion in the US and continues to grow, and these high medical expenses are impacting families and society [4]. Therefore, appropriate clinical applications to help older adults with knee OA to ameliorate their pain and restrictive movements and promote their QOL have become a matter of great clinical concern.

The progression of OA in the knee usually involves long-term changes. Although, surgical treatments can effectively improve the symptoms of knee OA, the risks and complications of surgery are higher in older adults [5]. For example, Nelson et al. [6] reported that compared to surgery and medical treatments, exercise is relatively safe for OA. Therefore, conservative approaches, including lifestyle modifications, medications, bracing, orthotics, and physical therapy interventions, might be generally recommended for reducing pain and improving the function for older people with knee OA. Indeed, several studies indicated that exercise can reduce pain and improve the function and health status of patients with knee OA [7, 8]. However, we found that older adults with knee OA do not prefer to do strengthening and aerobic exercises (low-speed running, hiking, and biking) in the clinic or follow home-based training protocols at home, because they feel that joint impacts and loading of the knees are increased, thereby inducing discomfort or pain of OA knees during and after exercising. Therefore, an ideal exercise for older adults with knee OA would entail lower joint impacts and loading of OA knee joints, which would increase the motivation for older adults with knee OA to perform exercise and help them enhance their physical performances in daily life [9].

Tai chi exercise is a multicomponent exercise and consists of slow movements, which might generate reduced joint impacts on the knee joint; it has become popular around the world [10]. Recent studies indicated that tai chi facilitates both physical function and psychological well being in older adults and people with neurological, rheumatologic, orthopedic, and cardiopulmonary diseases [11, 12], and the safety of tai chi has also been well-evaluated [13]. Furthermore, positive effects of tai chi exercise on degenerative OA were also evident in previous research [14, 15]. In a previous randomized controlled trial, the study showed that tai chi exercise three times a week for 24 weeks improved gait performance, balance, and muscle power of the lower extremities and reduced pain among older adults with knee OA [14]. A systematic review and meta-analysis study also indicated that tai chi has a tendency to improve walking and knee extensor strength, and ameliorate pain and stiffness in individuals with OA [15]. In addition, some researchers have indicated that tai chi produces beneficial effects similar to those of a standard course of physical therapy for knee OA. The primary outcome measurements for the tai chi interventions on knee OA are often pain scales and lower limb function. Despite several studies showing positive benefits of tai chi exercise on muscle strength, balance, and walking performance of the lower limbs, little is known about its effects on the comprehensive functional fitness of the upper and lower extremities in older adults with a knee OA diagnosis and how it impacts the overall functional performance for activities of daily living (ADLs). Functional fitness could reflect an older adult’s ability to perform physical ADLs with relative ease [16]. Each component of functional fitness declines with age, which negatively affects the QOF and reduces the confidence and motivation to engage in physical activities. This might lead to a sedentary lifestyle, accelerating the effects of aging [17]. Clinical research has indicated that functional fitness tests can be used to understand age-related health changes during the seniors’ aging process. Thus, implementing regular monitoring and predicting trends of aging in older adults has been suggested [18–20]. Recent research also showed that functional fitness tests could reflect different physical activity levels and demonstrate that seniors with high physical activity levels have better fitness performance [21]. Another previous study suggested that resistance exercises and balance training may have a crossover effect on the functional fitness of the elderly; however, cardiorespiratory fitness and flexibility may require specific interventions. The prolonged training duration increases the burden on the lower limbs of patients with OA, which affects exercise compliance. However, only a few studies have investigated tai chi’s effects on functional fitness in healthy young and old adults, and these studies showed conflicting results [22, 23]. Therefore, the purpose of this study was to investigate the impact of tai chi exercise on functional fitness in community-dwelling older adults with degenerative knee OA. We hypothesized that tai chi exercise can improve functional fitness in community-dwelling older adults with knee OA. We hypothesized that tai chi exercise can improve functional fitness in community-dwelling older adults with knee OA.

## Methods

### Study design

**Fig. 1:**
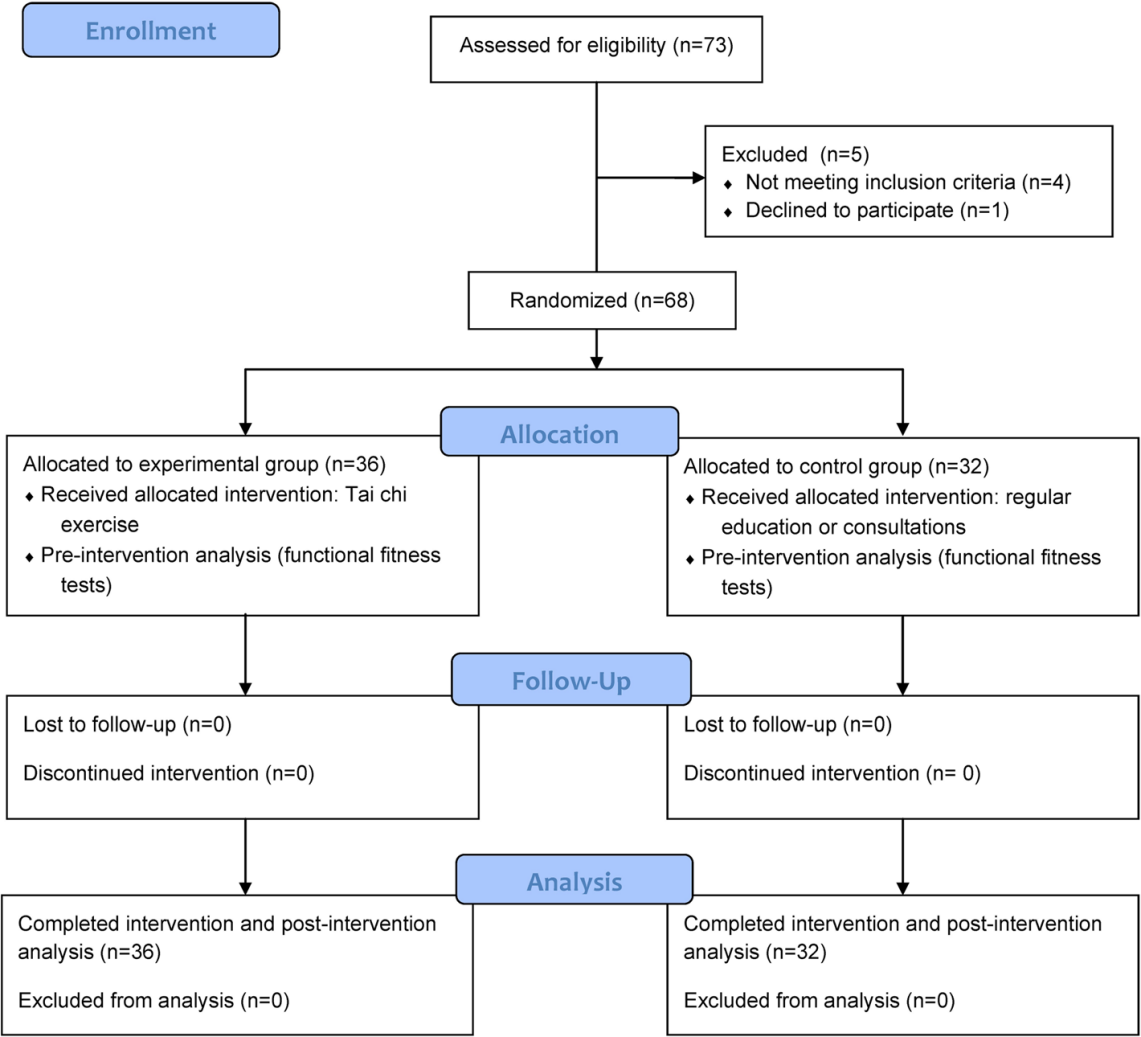
Flow diagram of the randomization procedure and outcome measurements of the study

This study was an intervention study of a single-blinded (the functional fitness evaluator was blinded when collecting data), randomized trial comparing tai chi exercise (the experimental group, TCE) and regular health education programs (the control group, CON) among older adults with mild knee OA (trial registry no. NCT03660254) and investigated the intervention impacts of tai chi exercise on functional fitness in community-dwelling individuals (Fig. 1). During the study period, a research assistant was responsible for monitoring and ensuring that all participants adhered to the tai chi exercise sessions and regular health education programs were delivered on time in both groups. The statistician analyzing functional fitness data was also blinded.

### Subjects

Based on the findings of a similar study on OA patients [24], the sample size requirement was computed via G*power using a medium effect size of 0.5, an ɑ of 0.05, and a power of 0.80. A minimum sample size of 34 participants was required. Furthermore, according to the results of a previous tai chi study on fitness gain in healthy old adults [25], 31 patients were enrolled in the 12-week TCE group. The TCE group showed a 25–34 % increase in strength compared with an increase of less than 10 % in the CON group. As for the timed up-and-go test, the TCE group experienced a 13 % decrease compared with the 7 % decrease in the CON group. We determined the minimal sample size to be 31 participants in each group. Seventy-three older adults with knee OA diagnosis from the local communities, complying with the inclusion and exclusion criteria, were invited to participate in the study via oral recruitment between January and October 2018 (this study was approved and valid from January 10, 2018, to January 9, 2019). Inclusion criteria were: (1) being aged ≥ 65 years, (2) meeting criteria of the American College of Rheumatology for mild knee OA, (3) with a Mini-Mental Status examination score of ≥ 25 and able to fluently use Chinese for communication, (4) able to stand without discomfort for more than 60 min, and (5) able to walk with no assistive devices. Exclusion criteria were: (1) a severe medical condition or pain and unable to do exercises for knee OA in the past year, (2) the presence of acute inflammation which would limit full participation during the study period, (3) the presence of a major cardiovascular or heart disease, (4) dizziness, vomiting, or difficulty breathing during exercise in the past year, or (5) an inability to understand and follow study instructions. However, we found that four subjects had mild inflammation and felt discomfort performing functional fitness tests; another subject stated that she could not follow the protocols. A total of five subjects dropped out of the study. Finally, 68 participants were enrolled in the study and were randomly assigned to the TCE (*n* = 36) and CON groups (*n* = 32). A flowchart of this study’s randomization and outcome measurements is presented in Fig. 1. Each participant signed an informed consent form before participation. The Institutional Review Board of the Taipei Medical University approved the study protocol (no. N201709036).

### Tai chi exercise program

Early studies reported that the Sun style of tai chi exercise is a slower style with gentler motions compared to other tai chi styles, which may reduce lower joint impacts and is more suitable for older adults with knee OA [26, 27]. Therefore, in this study, an experienced tai chi exercise instructor experienced in the Sun-style tai chi exercise protocols led these subjects with knee OA in the TCE group in performing Sun-style tai chi exercise in a 60-min exercise session (10 min of warm up, 40 min of the main tai chi program, and 10 min of cool down), twice a week for 12 weeks. In each session, the instructor taught these older adults tai chi exercise theory and how to use the mind-body relaxation techniques and procedures; warm-up and cool-down periods were also included during the exercise period.

### The education program

During the study period, all participants in the CON group received regular health education programs or consultations on self-care, self-health management, medication safety, fall prevention, and home-based exercise courses twice a week. Each session included 40 min of education courses and 20 min of home-based exercise. Specific staff, consisting of clinical nurses, pharmacists, physicians, social workers, and volunteers from local community development associations and health centers, delivered courses and met with the CON group in their community center for 1 h each session. Attendance was checked by staff members without any other interventions. A certified blinded functional fitness evaluator performed the functional fitness assessments and collected the data before and after the intervention. Throughout the study period, staff tracked the reasons for missed sessions and the number of missed sessions.

### Outcome measurements

Valid and reliable functional fitness tests were performed to indicate the comprehensive physiologic capabilities and assess the impacts of tai chi exercise on the improvement of the physical conditions required to perform ADLs in older adults with knee OA [18–20, 28, 29]. Functional fitness tests were performed before and after the 12-week intervention in both the TCE and CON groups, including the 30-s chair stand, 30-s arm curl, 2-min step, chair sit-and-reach, back-scratch flexibility, single-leg stand, functional reach, 8-foot up-and-go, and 10-m walk tests. Because tai chi consisted of lower intensity resistance exercises and balance components, the primary outcomes were strength (30-s chair stand and 30-s arm curl) and balance (chair sit-and-reach, single-leg stand, functional reach, and 8-foot up-and-go). Flexibility and endurance were the secondary outcomes.

### Statistical analysis

All data were entered into a computer by the investigator and analyzed with the Statistical Package of the Social Sciences (SPSS) vers. 18.0 statistical software (SPSS, Chicago, IL, USA). Demographic data of subjects are presented using descriptive statistics. If the data were continuous, an independent *t*-test test was used to compare between the two groups at the baseline. If the data were categorical, a Chi-squared test was performed to compare between the two groups. Several indicators of baseline functional fitness showed heterogeneity between the TCE and CON groups. Dependent t-tests were performed to analyze the differences between the pre-and post-tests within the two groups. Cohens’ d was used for effect size where the value indicates small (0.2), medium (0.5), and large (0.8) effects [30]. We performed an ANCOVA test to test the group effect of functional fitness in the post-test adjusted for age, sex, BMI, and pre-test score. The partial eta squared (η²) value of the ANCOVA tests indicates effect sizes in accordance with Cohen’s guidelines (0.01, small; 0.06, medium; and 0.14, large) [30]. Statistically significant differences ​​were set at *p* < 0.05.

## Results

Table 1 shows baseline characteristics of participants’ background and functional fitness in TCE and CON groups. Independent *t*-test results revealed that there were significant differences in the 2-min step test (*p* = 0.008), chair sit-and-reach test (*p* = 0.039), single-leg stand with eyes closed (*p* = 3.02*10^− 5^), functional-reach test (*p* = 0.002), and 8-foot up-and-go test (*p* = 0.049) between the two groups, indicating that CON had better functional fitness than the TCE group.

**Table 1:** Baseline demographic data and physical fitness values in the TCE and CON groups

Variable	CON group (*N* = 32)	TCE group (*N* = 36)	95 % Confidence interval		*t*	*p*	Cohen’ *d*
			Lower	Upper			
Age (years)	75.4 ± 6.4	77.4 ± 5.9	-	-	1.355	0.180	0.325
Sex (Female/Male)	30/2	32/4	-	-	-	0.481	-
Body-mass index (kg/m^2^)	24.0 ± 2.7	24.7 ± 2.6	-0.589	2.015	1.093	0.278	0.264
30-s arm curl test (no. of times)	23.3 ± 6.5	21.4 ± 5.8	-4.994	1.029	-1.315	0.193	0.308
30-s chair stand (no. of times)	17.3 ± 3.5	16.2 ± 3.7	-2.789	0.734	-1.165	0.248	0.305
2-min step test (no. of times)	170.6 ± 35.6	146.2 ± 37.4	-42.130	-6.675	-2.748	**0.008**	0.668
Chair sit-and-reach test (cm)	-4.4 ± 8.8	-9.1 ± 9.4	-9.076	-0.240	-2.105	**0.039**	0.516
Back-scratch flexibility test (cm)	-7.4 ± 6.1	-9.9 ± 12.1	-7.135	2.002	-1.088	0.281	0.261
Single-leg stand with eyes opened (s)	13.8 ± 12.9	12.8 ± 12.3	-7.156	5.039	-0.347	0.730	0.080
Single-leg stand with eyes closed (s)	5.3 ± 2.2	3.2 ± 1.6	-3.021	-1.159	-4.482	3.02*10^**− 5**^	1.092
Functional-reach test (cm)	20.5 ± 6.9	16.0 ± 4.6	-7.426	-1.664	-3.235	**0.002**	0.767
8-foot up-and-go test (s)	8.4 ± 3.2	10.0 ± 3.4	0.009	3.188	2.007	**0.049**	0.485
10-m walk test (s)	8.0 ± 1.8	8.3 ± 2.5	-0.788	1.331	0.512	0.611	0.138

Data are presented as the mean ± standard deviation

bold *p* value represents reaching the significant level (*p* < 0.05)

Table 2 reveals the dependent t-test results of the difference between the two groups in the pre- and post-tests. In the TCE group, there were significant differences between scores in the pre- and post-tests with a small to medium effect (Cohen’s d = 0.37 ~ 0.72), including the 30-s arm curl test (*p* = 0.005), 2-min step test (*p* = 3.19*10^− 4^), back-scratch flexibility test (*p* = 0.006), single-leg stand with eyes closed (*p* = 0.4.66*10^− 4^), functional-reach test (*p* = 0.046), and the 8-foot up-and-go test (*p* = 0.046). In the CON group, only two parameters did not show significant changes.

**Table 2:** Difference of pre-test and post-test functional fitness within group

Functional fitness parameter	CON group (***N***=32)				TCE group (***N***=36)			
	Mean difference (95% CI)		*p*	d	Mean difference (95% CI)		*p*	d
30-s arm curl test (no. of times)	-3.03	(-4.46, -1.60 )	1.33*10^**-4**^	0.72	2.13	(0.71, 3.54 )	**.005**	0.54
30-s chair stand (no. of times)	-4.61	(-5.69, -3.53 )	2.90*10^**-10**^	1.45	0.16	(-1.14, 1.45 )	.807	0.04
2-min step test (no. of times)	-24.89	(-34.06, -15.72 )	3.41*10^**-6**^	0.92	20.34	(10.10, 30.59 )	3.19*10^**-4**^	0.72
Chair sit-and-reach test (cm)	-3.79	(-5.71, -1.87 )	3.02*10^**-4**^	0.67	1.28	(-0.02, 2.57 )	.053	0.36
Back-scratch flexibility test (cm)	1.26	(-2.18, 4.70 )	.461	0.12	1.70	(0.51, 2.89 )	**.006**	0.52
Single-leg stand with eyes opened (s)	-20.69	(-30.38,-11.01 )	1.16*10^**-4**^	0.72	-3.21	(-8.51, 2.09 )	.226	0.22
Single-leg stand with eyes closed (s)	-1.97	(-2.97, -0.97 )	3.04*10^**-4**^	0.67	1.60	(0.77, 2.43 )	4.66*10^**-4**^	0.69
Functional-reach test (cm)	-4.97	(-6.61, -3.34 )	4.68*10^**-7**^	1.03	1.67	(0.03, 3.32 )	**.046**	0.37
8-foot up-and-go test (s)	1.98	(1.29, 2.67 )	1.26*10^**-6**^	0.97	-1.33	(-2.10, -0.56 )	**.001**	0.62
10-m walk test (s)	0.06	(-0.47, 0.59 )	.816	0.04	0.02	(-0.45, 0.49 )	.936	0.01

*CI* confidence interval

bold *p* value represents reaching the significant level (*p*<.05)

Table 3 demonstrates the ANCOVA results of differences in functional fitness between the TCE and CON groups after adjusting for age, sex, BMI, and pre-test scores. The results revealed that participants’ functional fitness in TCE had significantly higher adjusted mean scores in the post-tests than that in the CON group, including the 8-foot up-and-go (*p* = 2.39*10^− 7^), 2-min step (*p* = 7.08*10^− 7^), 30-s chair stand (*p* = 6.96*10^− 7^), functional reach (*p* = 4.72*10^− 6^), single-leg stand with eyes closed (*p* = 2.74*10^− 5^), chair sit-and-reach (*p* = 0.001), and single-leg stand with eyes opened (*p* = 0.002) tests. Tai chi intervention produced significant improvements in several indicators of functional fitness, which all showed large effect sizes (η²=0.16 ~ 0.34). Compared to the CON group, the participants in the TCE group showed better functional fitness performance after the intervention. Therefore, tai chi had a large positive effect on functional fitness in older adults.

**Table 3:** ANCOVA results of functional fitness between the groups

Functional fitness parameter	CON group (*N*=32)		TCE group (*N*=36)		Adjusted post-test mean difference between groups		*F*	*p*	η²
	Mean	SE	Mean	SE	Mean difference (95% CI)				
30-s arm curl test (no. of times)	20.39	0.71	25.13	0.67	4.75	(2.76, 6.73 )	22.74	1.11*10^**-5**^	0.26
30-s chair stand (no. of times)	16.60	0.61	21.27	0.57	4.66	(2.97, 6.36 )	30.31	6.96*10^**-7**^	0.32
2-min step test (no. of times)	141.75	4.74	178.69	4.45	36.94	(23.53, 50.36 )	30.26	7.08*10^**-7**^	0.32
Chair sit-and-reach test (cm)	-7.55	0.79	-3.61	0.74	3.93	(1.72, 6.15 )	12.62	**.001**	0.16
Back-scratch flexibility test (cm)	-9.88	1.22	-10.49	1.15	-0.61	(-3.97, 2.76 )	0.13	.720	0.00
Single-leg stand with eyes opened (s)	16.72	3.91	33.79	3.68	17.07	(6.29, 27.85 )	10.01	**.002**	0.14
Single-leg stand with eyes closed (s)	2.65	0.52	6.09	0.49	3.44	(1.92, 4.97 )	20.42	2.74*10^**-5**^	0.24
Functional-reach test (cm)	16.87	0.82	22.73	0.77	5.86	(3.52, 8.20 )	25.01	4.72*10^**-6**^	0.28
8-foot up-and-go test (s)	10.41	0.36	7.49	0.34	-2.92	(-3.93, -1.91 )	33.42	2.39*10^**-7**^	0.34
10-m walk test (s)	8.07	0.23	8.14	0.22	0.07	(-0.56, 0.71 )	0.06	.815	0.00

All mean, stand error, mean difference presents the post-test score adjusted for age, sex, BMI, and pre-test scores

*CI* confidence interval

bold *p* value represents reaching the significant level (*p*<.05)

## Discussion

Because of knee discomfort and stiffness in patients with degenerative arthritis of the knee, their ability of walking and performing physical activities are often encumbered. Traditional strengthening exercises (running, hiking, and biking exercises) with higher joint impacts and loading might not suit patients with knee OA, especially older patients. A recent study also suggested that knee OA patients with pain or physical disability of the affected joints are less likely to initiate and follow clinician-prescribed exercise programs, resulting in poor exercise adherence and physical condition [31]. In contrast, tai chi exercise is a mild-to-moderate intensity and relatively slower exercise. It may generate more lower joint impact and loading of OA knees than traditional exercises, and hence it is appropriate for implementation in older patients [11]. Our results showed that tai chi exercise could significantly enhance comprehensive functional fitness, including muscle strength, endurance, balance performance, and lower limb flexibility, which may indirectly enhance the daily life functions and self-care abilities of older adults with knee OA.

### Impact of tai chi exercise on muscle strength, endurance, and flexibility in older adults with knee OA

Upon completing 12 weeks of tai chi exercise twice a week, the TCE group experienced significantly higher improvement in the 30-s arm curl, 30-s chair stand, and 2-min step tests than the CON group of older adults with knee OA. This meant that the upper and lower extremities’ muscle strength and endurance improved after tai chi exercise. Our findings are consistent with previous studies and indicate that after tai chi exercise programs, muscle strength and endurance increase in older adults and patients with neurological, rheumatologic, orthopedic, and cardiovascular diseases [11, 32]. Additionally, the results showed that subjects’ lower extremities flexibility (chair sit-and-reach test) was better after the tai chi exercise intervention than that of the CON. However, the upper extremities’ flexibility did not significantly differ between the two groups. The lower extremities increased flexibility could result from the repetition of multiple joint movements in slow, whereas the stretching of the soft tissues and working on the range of motion extension of the upper limbs were performed less. Similar findings were also reported in a recent study on the effect of a modified tai chi Qigong intervention on functional fitness in the elderly [31]. Some previous studies have shown controversial results regarding the effects of tai chi on older adults, possibly because the healthy elderly people in their studies had a better physical fitness baseline, making it difficult to improve in tai chi training. For healthy subjects, other training programs have been shown to improve balance and functional fitness [22].

### Changes in the balance and functional performances before and after the tai chi intervention in older adults with knee OA

Our results revealed that after the tai chi exercise intervention, the balance and functional performances were significantly improved, such as single-leg stand with eyes opened and eyes closed, functional reach, and 8-foot up-and-go were better in TCE group than in the CON. Our results demonstrated that the balance benefits were similar to those in a previous study, where the balance improved after 12 weeks of a tai chi exercise intervention in older adults with cardiovascular disease [32]. Because of enhanced balance performance, tai chi exercise’s effects on the risk of falling improved likewise. For example, Lomas-Vega et al. [33] reported protective effects of tai chi exercise on fall incidence in both short and long term from high-quality evidence of a meta-analysis study. However, in the 10-m walking test results, we found no significant improvement in the TCE group compared to theCON. This could be the reason that the 10-m walk test focuses on evaluating functional performance requiring speed. Tai chi is a relatively slower exercise compared to the isokinetic exercises performed in the clinic and might not directly enhance gait speed performance.

### Improvements in psychosocial status after tai chi exercise

Besides improvements in the functional fitness area, tai chi exercises may enhance mental capacity and psychosocial conditions in older adults’ daily activities with knee OA. For example, recent studies pointed out that tai chi exercise positively affects health-related quality of life, psychological well-being (stress, anxiety, and depression), and social networking of older adults and people with diseases [11, 12, 34]. In this study, we found that several subjects lived alone and reported that they not only had better physical fitness and mental health conditions but were also more likely to participate in social activities after tai chi exercise intervention. This potential psychosocial benefit could be explained by tai chi being a group exercise and therefore reinforcing psychological health and social interconnection, improving the QOL of older adults [12, 35].

### Study limitations

In the current study, it was difficult to check how the CON group did in their daily lives as their programs were recorded and traced objectively only during meetings twice per week. We only ensured that they did not have extra consultations during the research period. Meanwhile, although the psychosocial benefit had been reported for those patients who lived alone in this study, which was similar to a recent study also indicated that exercise could improve mental health and psychosocial conditions in older adults [36]. Further studies should be conducted to collect and assess cognitive and psychosocial health data using questionnaires and assessment scales and analyze the impacts of tai chi exercise on older adults’ psychosocial health status with knee. The most common pain evaluation tool for people with OA knee is the Western Ontario and McMaster Universities Osteoarthritis Index (WOMAC), which can be used to assess the positive effects of exercise programs focused on decreasing pain in patients with OA knee and other orthopedic diseases [11, 24, 37]. However, we did not apply this tool in our study, and we based our data only on patients’ reports. Although no subject reported pain or discomfort in the OA knees during or after the tai chi exercise program, this is objective information and presents a bias in understanding the real pain condition of patients with OA. We recommend that future research be conducted using WOMAC to provide quantitative data and reveal the changes in the pain perception of patients with knee OA during tai chi exercise intervention in the future. Additionally, this study cooperated with the local community and recruited participants in the CON group, all of whom were asked to attend weekly regular health education programs in the local community, such as self-care, self-health management, fall prevention, and home-based muscle strength exercise courses carried out by nurses, pharmacists, physicians, social workers, and volunteers from local community development associations and health centers. All program information was reported by volunteers from this community with no log record of the frequency of intervention delivery. This should be considered because some of the regular health education programs might impact adherence and the benefits of functional fitness in older adults compared to the TCE group, warranting further analysis in future studies.

## Conclusions

This study indicated that tai chi exercise had positive effects on improving the functional fitness of older adults with mild knee OA, and this appropriate exercise approach might be helpful in improving the overall QOL of community-dwelling older adults with mild degenerative knee OA. Based on our findings and participants’ feedback in this study, we believe that tai chi exercise is an appropriate and complementary approach which is easily carried out by older adults with knee OA in community care centers and long-term care institutions, because this exercise consists of slow movements with lower joint impacts and loading, is a safe, simple, and low-cost exercise, and also helps participants build social connections.

## Data Availability

All data used and analyzed in this study are available from the corresponding author on reasonable request.

## Abbreviations

OA: Osteoarthritis
QOL: Quality of life
ADLs: Activities of daily living
CI: Confidence interval
SPSS: Statistical Package of the Social Sciences
ANCOVA: Analysis of covariance
WOMAC: Western Ontario and McMaster Universities Osteoarthritis Index
